# Substitution of D-Arginine at Position 11 of α-RgIA Potently Inhibits α7 Nicotinic Acetylcholine Receptor

**DOI:** 10.3390/md21060326

**Published:** 2023-05-26

**Authors:** Yong Wu, Junjie Zhang, Jie Ren, Xiaopeng Zhu, Rui Li, Dongting Zhangsun, Sulan Luo

**Affiliations:** 1 School of Medicine, Guangxi University, Nanning 530004, China; zjjstudy@163.com (J.Z.); hndx2303@163.com (J.R.); zhuxiaopeng@gxu.edu.cn (X.Z.); zhangsundt@163.com (D.Z.); 2 Shanghai Institute of Materia Medica, Chinese Academy of Sciences, Shanghai 201203, China; flychckn@163.com; 3 Key Laboratory of Tropical Biological Resources of Ministry of Education, Hainan University, Haikou 570228, China

**Keywords:** conotoxin, peptide synthesis, CD structure, electrophysiology, nicotinic acetylcholine receptors

## Abstract

Conotoxins are a class of disulfide-rich peptides found in the venom of cone snails, which have attracted considerable attention in recent years due to their potent activity on ion channels and potential for therapeutics. Among them, α-conotoxin RgIA, a 13-residue peptide, has shown great promise as a potent inhibitor of α9α10 nAChRs for pain management. In this study, we investigated the effect of substituting the naturally occurring L-type arginine at position 11 of the RgIA sequence with its D-type amino acid. Our results indicate that this substitution abrogated the ability of RgIA to block α9α10 nAChRs, but instead endowed the peptide with the ability to block α7 nAChR activity. Structural analyses revealed that this substitution induced significant alteration of the secondary structure of RgIA[11r], which consequently affected its activity. Our findings underscore the potential of D-type amino acid substitution as a promising strategy for designing novel conotoxin-based ligands targeting different types of nAChRs.

## 1. Introduction

Nicotinic acetylcholine receptors (nAChRs) are members of the Cys-loop ligand-gated ion channel superfamily which play crucial roles in rapid synaptic transmission and have been implicated in a range of nervous system diseases. To date, sixteen nAChR subunits (α1–α7, α9, α10, β1–β4, γ, δ and ε) have been identified in mammalian species. These subunits combine into hetero- or homo-pentamers to form nAChRs with different pharmacological and kinetic properties, as well as their localization [1]. The homomeric α7 nAChR has been intensely studied since its original discovery [2]. In the central nervous system, α7 nAChR is mainly distributed in the hippocampus and the cerebral cortex, regions associated with learning and memory mechanisms. Major human pathologies such as epilepsy, myasthenic syndromes, schizophrenia, Parkinson’s and Alzheimer’s diseases result from a dysfunction of α7 nAChR. Moreover, α7 nAChR is also located on the surface of macrophages, which plays a vital role in the cholinergic anti-inflammatory pathway. Therefore, ligands specifically designed to target the α7 receptor have the potential to be developed as drugs for the treatment of these diseases [3].

Conotoxins are a class of disulfide-rich peptides that are present in the venom of cone snails. Conotoxins have been shown to selectively and effectively modulate the function of ion channels and receptors in the nervous system [4]. Some of these peptides have been utilized as pharmacological probes, while others have been developed as potential drug leads. Notably, ω-conotoxin MVIIA, which is known as Ziconotide or Prialt, is a well-known example of a conotoxin-derived drug that targets the calcium channel CaV_2.2_ and is approved for the treatment of neuropathic pain [5,6]. Among the families of conotoxins, α-conotoxins are distinguished by their selective antagonism against different subtypes of nAChRs, rendering them a significant source for the development of nAChR ligands. The α-conotoxin family consists of peptides comprising 12–19 amino acids, which typically possess an amidated C-terminus, wherein the hydroxyl group of the carboxyl group is replaced with an amide. These peptides contain four cysteines (CysI-CysIII, CysII-CysIV), forming two pairs of disulfide bonds. Based on the number of amino acids between the cysteines (m/n), they can be categorized into various subtypes. In general, 3/5 (m/n) α-conotoxins act as selective antagonists of muscle type, whereas 4/7, 4/4 and 4/3 α-conotoxins selectively antagonize non-muscle type nAChRs. The pharmacological effects of these peptides have been extensively investigated. Studies have demonstrated that the first loop harbors a conserved hydrophobic region, which determines its binding, whereas the second loop comprises a more variable region that confers selectivity. In vivo and in vitro, some of these peptides display therapeutic potential. Notably, some conotoxins have been found to inhibit the α7 receptor with high affinity [4,7,8,9].

**Figure 1:**
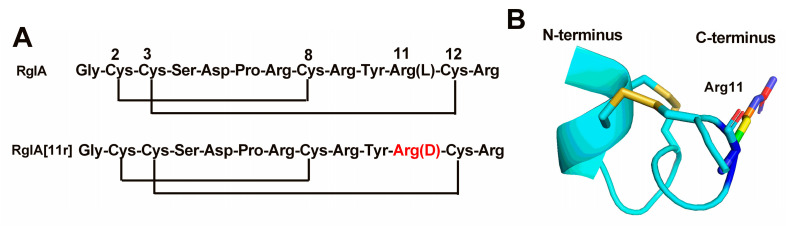
Sequences of conotoxins RgIA and RgIA[11r] and the three-dimensional structure of RgIA. (**A**) Amino acid sequences of RgIA and RgIA[11r] with two disulfide bonds. (**B**) The structure of RgIA arises from its interaction with the extracellular domain of the α9 nAChR subunit (PDB: 6HY7) [14].

α-RgIA was discovered through cDNA sequencing of the venom gland of *Conus regius* [10]. The sequence and structure of this peptide are depicted in Figure 1. It belongs to the relatively uncommon class of α4/3-conotoxins, featuring four cysteines that form two pairs of disulfide bonds in the sequence and a high abundance of arginine residues. RgIA and its derivatives selectively and potently inhibit heterologous α9α10 nAChR, which was found to be a neuropathic pain target. RgIA4, an analogue of RgIA, has garnered attention due to its high affinity towards human α9α10 nAChRs [11]. The University of Utah has licensed RgIA4 to Kineta, under the designation KCP-400, for preclinical development [12]. However, the high arginine content of RgIA has raised concerns regarding its stability and in vivo bioavailability. To address this issue, we have employed a strategy of substituting amino acids with their D-form counterparts, leading to improved stability of RgIA while preserving its biological activity [13]. In this study, we have identified that one of the mutants containing a D-type amino acid substitution, RgIA[11r] (Figure 1), effectively inhibits the α7 nAChR and significantly decreases the potency at the α9α10 nAChR. These results suggest that D-type amino acid substitution not only enhances the stability of conotoxins but also enables the design of peptide ligands that can target other nAChR subtypes.

## 2. Results

### 2.1. Synthesis of RgIA and RgIA[11r]

**Figure 2:**
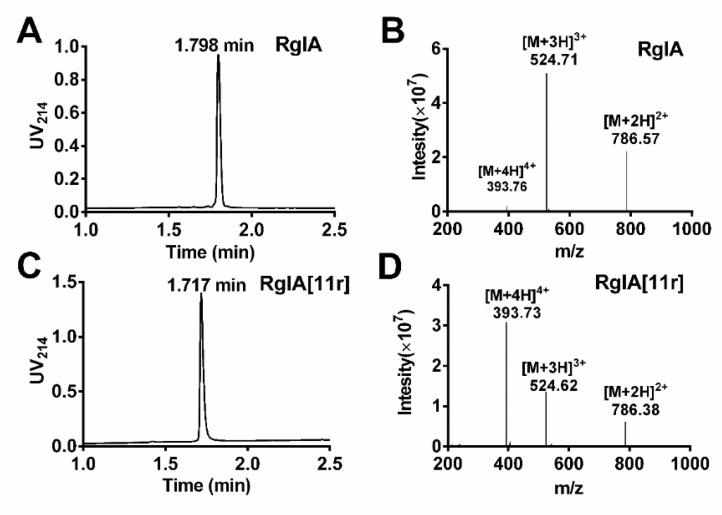
Analytical RP-UPLC profiles and ESI-MS spectra of α-CTxs RgIA and RgIA[11r]. (**A**) RP-UPLC chromatogram of RgIA; (**B**) Electrospray ionization mass spectrometry (ESI-MS) data for RgIA with the observed monoisotopic mass of 1571.13 Da; (**C**) RP-UPLC chromatogram of RgIA[11r]; (**D**) ESI-MS data for RgIA[11r] with an observed monoisotopic mass of 1570.92 Da.

This study employed the Fmoc solid-phase synthesis method to synthesize linear peptides of RgIA and RgIA[11r]. To produce RgIA[11r], the L-Arg residue at position 11 was substituted with D-Arg, yielding two peptides with identical theoretical molecular weights. To synthesize the folded peptides, a directed two-step folding approach was utilized. The Cys pairs were orthogonally protected using S-trityl (S-Trt) and acid-stable S-acetamidomethyl (S-Acm) groups. Selective removal of the S-trityl groups was achieved through cleavage from the resin, allowing the deprotected Cys residues to be oxidized with 20 mM potassium ferricyanide and 0.1 M Tris-HCl. Semi-preparative reversed phase–high-performance liquid chromatography (RP-HPLC) purification of the monocyclic peptide was then carried out, followed by treatment with iodine, resulting in the formation of the second disulfide bond between Cys3-Cys12. The molecular mass and purities of all peptides were validated with electrospray ionization–mass spectrometry (ESI-MS) and analytical RP-UPLC, respectively, as illustrated in Figure 2. The retention time of RgIA was 1.798 min, whereas that of RgIA[11r] was 1.717 min, indicating that RgIA[11r] was more hydrophilic when position 11 was substituted with D-Arg. The molecular weight of RgIA was determined to be 1571.13 Da, and that of RgIA[11r] was 1570.92 Da, consistent with the theoretical molecular weight. Our study demonstrates the successful synthesis and purification of folded RgIA and RgIA[11r] peptides using a directed two-step folding approach.

### 2.2. Potency of RgIA and RgIA[11r] at the Different Types of nAChRs

**Figure 3:**
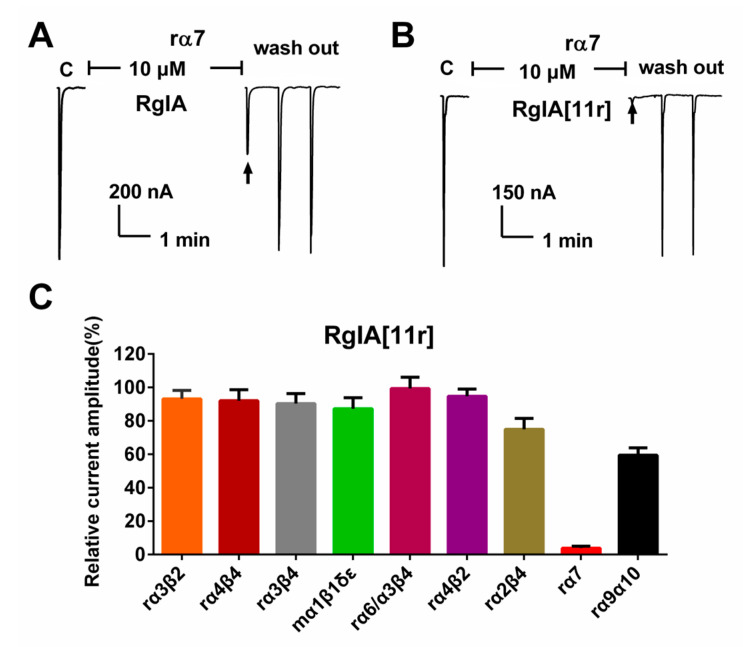
Inhibitory activity of RgIA and RgIA[11r] at different types of nAChRs. (**A**) Representative traces of ACh-evoked currents mediated by rα7 nAChRs in the presence of 10 μM RgIA. (**B**) Representative traces of ACh-evoked currents mediated by rα7 nAChRs in the presence of 10 μM RgIA[11r]. (**C**) Bar graph showing the inhibition of ACh-evoked peak current amplitude mediated by α3β2, α4β4, α3β4, mα1β1δγ, α6/α3β4, α4β2, α2β4, α7 and α9α10 nAChRs by RgIA[11r] (10 μM). Data points represent mean ± SEM (*n* = 3–6).

We conducted a comprehensive assessment of RgIA[11r]’s inhibitory activity (10 μM) against various types of nAChRs, namely rat (r) α3β2, α4β4, α3β4, α6/α3β4, α4β2, α2β4, α7 and α9α10; and mouse (m) α1β1δγ, heterologously expressed in *Xenopus laveis* oocytes, using the two-electrode voltage clamp recording method. The results are presented in Figure 3. At α7 nAChRs, wild-type RgIA (10 μM) demonstrated a 60% inhibitory effect on acetylcholine (ACh)-evoked currents. In contrast, RgIA[11r] displayed potent activity at α7 receptors, inhibiting approximately 95% of the ACh currents. Inhibition of other receptors was below 50% (Figure 3C). Remarkably, previous studies have reported that RgIA inhibits α9α10 at nanomolar levels [13]. However, surprisingly, 10 μM RgIA[11r] only inhibited 60% of α9α10 ACh currents.

**Figure 4:**
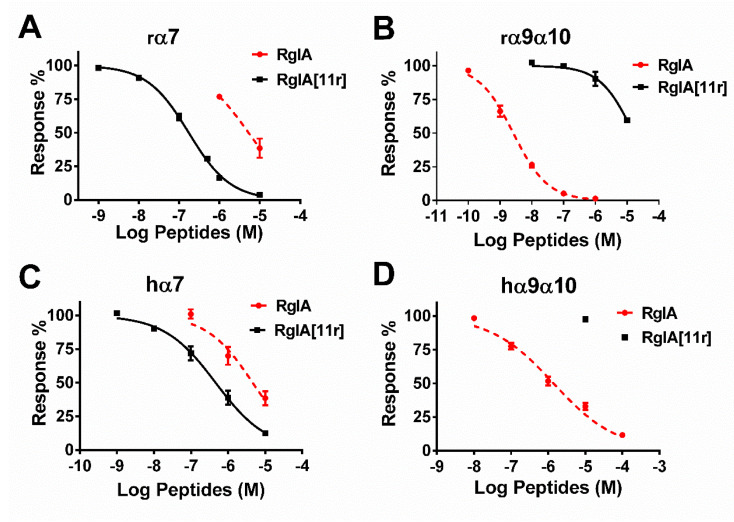
Concentration–response curves for the relative amplitude of ACh-evoked currents by RgIA and RgIA[11r] at different types of nAChRs; (**A**) rat α7, (**B**) rat α9α10, (**C**) human α7 and (**D**) human α9α10. Data are presented as mean ± SEM from 3 to 11 independent oocyte experiments.

The effective inhibition of hα7 nAChRs by RgIA at 10 μM having been established, we proceeded to determine the concentration response relationships of RgIA and RgIA[11r] at α7 and α9α10 nAChRs (Figure 4). The corresponding half-maximal inhibitory concentration (IC_50_) values for their inhibition of ACh-evoked currents mediated by rat α7 and α9α10 nAChRs are summarized in Table 1. Wild-type RgIA displayed a robust antagonistic activity against rα9α10 receptors, exhibiting an IC_50_ value of 2.6 nM, but a weak activity against rα7, with an IC_50_ of only 5313 nM, a result similar to those obtained previously [10]. In the present study, the replacement of the L-type arginine at position 11 with D-arginine resulted in the reversal of the activity of the mutant RgIA[11r]. RgIA[11r] showed inhibitory activity against rα7 with an IC_50_ of 163 nM, while it exhibited relatively minimal (IC_50_ = 15,820 nM) against rα9α10. Consequently, we proceeded to determine the inhibitory activity of RgIA and RgIA[11r] against human α9α10 and α7, and the results are presented in Figure 4 and Table 1. Wild-type RgIA displayed weak activity against both human-derived α9α10 and α7 receptors, exhibiting IC_50_ values of 1398 and 4608 nM, respectively. Surprisingly, RgIA[11r] showed no detectable activity against hα9α10, but still maintained a potency of 463 nM against hα7. Overall, compared to the wild-type RgIA, RgIA[11r] demonstrated a significant enhancement in activity towards rat and human α7 nAChRs, with an increase of 32.6-fold and 9.96-fold, respectively. However, its activity towards α9α10 nAChRs was nearly completely lost. These findings suggest that the conformation of the arginine residue at position 11 of RgIA was altered, with significant effects on its inhibition at α7 and α9α10 nAChRs.

### 2.3. Circular Dichroism (CD) Spectra of RgIA and RgIA[11r]

**Figure 5:**
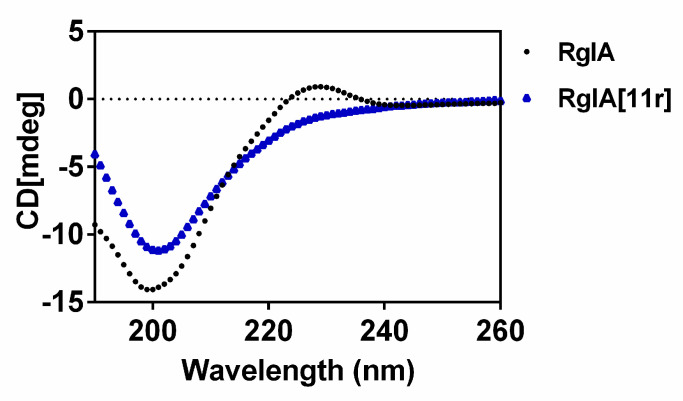
Circular dichroism spectra of RgIA and RgIA[11r].

In this study, we compared the secondary structure conformation of RgIA with that of RgIA[11r] using CD spectroscopic analysis (Figure 5). The 3D structure of RgIA is shown in Figure 1B, with a type I β-turn at its N-terminus from Cys2 to Asp5, while loop II from Tyr10-Cys12 is less well-defined. Our results show that when L-Arg residue at position 11 was replaced with a D-Arg residue, its negative absorption peak at 208 nm and its positive absorption peak at 222 nm were enhanced. This suggests that there may be a tendency for the formation of α-helix at Loop II of RgIA[11r], which indicates that the structure of RgIA is altered when the arginine of 11 is replaced by D-type amino acid. This result is significantly different from our previous study, where the replacement of Arg-13 by D-Arg had less effect on the structure of RgIA [13].

### 2.4. Serum Stability of RgIA and RgIA[11r]

**Figure 6:**
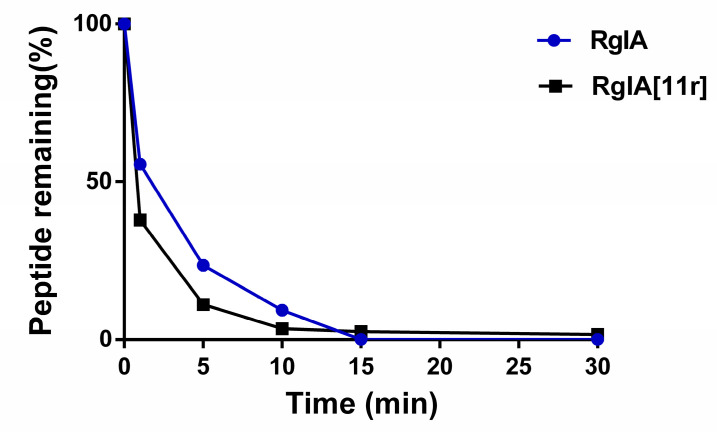
Serum stability of RgIA and RgIA[11r]. Error bars represent the mean ± SEM (*n* = 6).

We assessed the stability of wild-type RgIA and its 11-position D-amino acid substitution mutant, RgIA[11r], in human serum. The experimental results, as depicted in Figure 6, indicated poor stability for both peptides, with complete degradation observed within 30 min. This suggests that the substitution of individual amino acids with D-amino acids did not significantly enhance peptide stability. Our previous research has demonstrated a notable improvement in stability when all arginine residues were replaced with D-amino acids [13].

### 2.5. Molecular Docking (MD) Demonstrates Altered Potency between RgIA[11r] and α7 nAChRs

**Figure 7:**
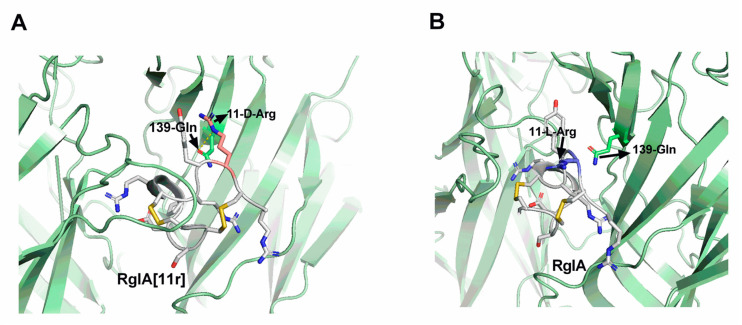
Molecular docking of RgIA/RgIA[11r] with hα7 nAChRs. (**A**) Molecular docking of RgIA[11r] with hα7 nAChRs, demonstrating the formation of hydrogen bonds between 11-D-Arg and the neighboring residue 139-Gln of the α7 nAChR. (**B**) Molecular docking of RgIA with hα7 nAChR, indicating the absence of interactions between 11-L-Arg and other residues of the α7 nAChR. The images were generated using PyMOL.

To further analyze the molecular mechanism underlying the targeting of the α7 nAChRs by RgIA[11r], we performed separate molecular docking studies between RgIA and RgIA[11r] with the human α7 nAChRs. The docking results, as illustrated in Figure 7, revealed that the 11-position D-amino acid in RgIA[11r] formed a hydrogen bond with residue 139-Gln in the extracellular region of the α7 nAChRs, while RgIA itself did not exhibit any interactions with the α7 nAChRs. The docking scores indicated a value of −10.8 kcal/mol for RgIA[11r] and −9.9 kcal/mol for RgIA when docked with the α7 nAChRs, suggesting an increased ligand affinity following the D-amino acid substitution mutation.

## 3. Discussion

Initially, we used D-alanine scanning to study the key amino acid residues of RgIA and found that, except for arginine at position 13, the activity was greatly reduced after replacing the amino acids at other positions with D-amino acids [13]. Based on the synthesized D-type RgIA derivatives, we tested the activity of other subtypes of nAChRs and were surprised to find that RgIA[11r] had strong inhibition activity against the α7 nAChRs. Building on this finding, we discovered that RgIA[11r] had strong blocking activity against human and murine α7 nAChRs.

Previous research has indicated that homomeric α7 nAChRs play a significant role in regulating neuropsychiatric and neurological disorders, as well as the inflammatory response of immune cells [15,16]. Given their potential as targets for treating inflammatory and neuropathic pain, the crucial physiological functions of α7 nAChRs have sparked considerable interest in developing drugs that target these receptors. Agonists or antagonists of α7 have emerged as promising candidates for drug development to treat α7-related diseases [2]. Numerous α-conotoxins and their analogues that can inhibit α7 nAChRs have been discovered (Table 2). Among these, α4/3-conotoxins ImI and ImII have been extensively studied and found to block various nAChR subtypes. In the case of α7 nAChRs, different research groups have reported varying potencies [17,18]. Multiple α4/7-conotoxins, such as GID, OmIA and PeIA, inhibiting α7 nAChRs with high potency, have demonstrated blocking effects on different types of nAChRs, with particularly strong activity against α3β2 nAChRs. The ArIB mutant ArIB [V11L, V16A] has potent activity targeting α7 nAChRs, with IC_50_s as low as picomolar levels, and a certain inhibitory effect at rα6/α3β2β3 nAChRs. In our laboratory, the α4/4-conotoxin [Q1G,ΔR14]LvIB had strong activity at rat α7 nAChRs, but low activity against human receptors [19]. In this study, RgIA[11r] exhibited an IC_50_ of 463 nM and 163 nM at human and rat α7 nAChRs, respectively. In comparison to other α-conotoxins acting on α7 nAChRs, RgIA[11r], although not the most potent, displayed relatively good selectivity for the α7 subtype with negligible inhibition at other nAChR subtypes. It is known that certain conotoxin peptides exhibit high activity against murine receptors but weak activity against human receptors, thereby limiting their clinical potential. The present investigation demonstrated that RgIA[11r] also exhibited higher activity against hα7 nAChRs, and therefore holds significant medicinal value. It is plausible that this peptide may serve as a framework for designing more potent peptide ligands targeting hα7 nAChRs.

Amino acids exist as two enantiomers: L and D, except for glycine, which lacks a chiral center. Although the D-amino acid (D-AA) enantiomer of α-amino acids was once considered unimportant in biological systems, it has garnered significant interest among researchers due to its ability to improve protein stability. One of the major challenges in peptide development is their susceptibility to degradation by proteases, resulting in a short half-life in the human intestine, plasma and cells. Therefore, enhancing peptide stability and bioavailability is crucial to improve their medicinal value. To address this challenge, analogues such as cyclodextrin (D)-amino-acid-engineered analogues have been designed to prolong their half-life in humans [30]. It is well recognized that the conversion of one enantiomer to another in a biological system can cause significant structural changes in peptides or proteins, which can affect their function and biological activity. The inversion of stereochemistry at the chiral center and the corresponding conformational preferences of D-AAs to their L-counterparts often lead to instability of the secondary structure when D-AAs are incorporated into L-peptides or proteins. However, it has been observed that peptides with α-helix, long β-strand and long loop structures are generally less sensitive to substitution with D-AAs than short β-strands [31]. In this study, we observed changes in the structure when arginine at the 11th position was replaced with the D-form, as determined by CD spectra. Although the amino acid sequence of conotoxin peptides is short, their spatial structure is compact, and the amino acids located in the loop formed by cysteine residues have a significant impact on their activity and selectivity. In the present investigation, the substitution of the 11th arginine residue with a D-configured amino acid resulted in the loss of the peptide’s original activity and a shift towards antagonizing α7 nAChRs. We utilized molecular docking to elucidate the molecular mechanism of interaction between the mutant RgIA[11r] and hα7 nAChRs. Our findings reveal the formation of new hydrogen bonds. However, our previous studies have demonstrated that when other residues except for Arg-11 in RgIA were substituted with D-configuration, no activity towards hα7 nAChRs was observed [13]. This suggests that the substitution of peptides with D-configured amino acids requires careful consideration and further investigation. Nevertheless, this study provides a direction for the subsequent design highly selective and active conotoxins.

## 4. Materials and Methods

### 4.1. Materials

Clones of rat (r)α2, α3, α4, α7 and β2, β3 and β4, as well as mouse (m) α1, β1, δ and ε cDNAs were generously provided by S. Heinemann (Salk Institute, La Jolla, CA, USA). It is worth noting that that the rα6 subunit is difficult to express in vitro, so we constructed the rα6/α3 chimeric subunit instead, which consisted of the N-terminal extracellular ligand-binding domain of the rα6 subunit and the remainder as the rα3 subunit segment. The rα6/α3 chimera clone was generously provided by J. E. Garrett (Cognetix, Inc., Salt Lake City, UT, USA). Clones of rα9 and rα10 were kindly provided by A.B. Elgoyen (Instituto de Investigaciones en Ingeniería Genética y Biología Molecular, Buenos Aires, Argentina). C. W. Luetje (University of Miami, Miami, FL, USA) provided clones of rβ2 and rβ3 subunits in the high-expressing pGEMHE vector. The RNAs of human α7, α9 and α10 nAChRs were synthesized using mMessage mMachine transcription kit (Ambion, Forster City, CA, USA). The mMESSAGE mMACHINE in vitro Transcription Kit and an RNA MEGA Clear Kit were purchased from Thermo Fisher Scientific (Austin, TX, USA). Acetylcholine chloride, atropine and bovine serum albumin (BSA) were obtained from Sigma (St. Louis, MO, USA). Acetonitrile (ACN, HPLC grade) was purchased from Thermo Fisher Scientific (Pittsburgh, PA, USA). Trifluoroacetic acid (TFA) was purchased from Tedia Company (Fairfield, OH, USA). Vitamin C (VC), (K_3_[Fe(CN)_6_]), I_2_ and other reagents were purchased from Guangzhou Chemical Reagent Company (Guangzhou, China). All standard amino acids, and preloaded resin for peptide synthesis, were purchased from GL Biochem (Shanghai, China). Side-chain protection for the following amino acids was as follows: L-Arg and D-Arg, 2,2,4,6,7-pentamethyl-dihydrobenzofuran-5-sulfonyl (Pbf); Ser, Tyr, tert-butyl (tBu); Cys, acetamidomethyl (Acm) and Cys, trityl (Trt). All other chemicals were analytical-grade and were obtained from Sigma. Reverse-phase (RP) HPLC analytical Symmetry Shield RP18 column (5 µm, 4.6 × 150 mm, 130-Å pore size), RP-UPLC ACQUITY UPLC BEH C18 column (1.7 µm, 2.1 × 50 mm, 130-Å pore size) and preparative XBridge Peptide BEH C18 column (5 µm, 19 × 100 mm, 130-Å pore size) were obtained from Waters Corp. (Milford, MA, USA). The female *Xenopus laevis* used for experiments were obtained from Nasco (Fort Atkinson, WI, USA) and were housed at 17 °C in our laboratory animal room and fed twice a week. All animal experiments were conducted in accordance with ethical standards for animal research (GXU-2023-0060).

### 4.2. RgIA and RgIA[11r] Synthesis

The synthesis of conopeptides was accomplished on a 0.05 mmol scale using a Liberty Blue automated peptide synthesizer (CEM, Charlotte, NC, USA), applying standard solid-phase Fmoc (9-fluorenylmethyloxycarbonyl) protocols using Fmoc-Arg(Pbf) Wang resin (0.49 mmol/g load). During the assembly process, Cys3 and Cys12 were integrated into the peptide chain with Acm side chain protection to aid in regioselective disulfide formation, while Cys2 and Cys8 were protected with Trt side chain. The peptides were subsequently cleaved from the resin with trifluoroacetic acid (TFA) in the presence of tri-isopropylsilane (TIPS) and water (9:0.5:0.5 (*v*/*v*/*v*) TFA:TIPS:water) at room temperature for 2 h, resulting in simultaneously removing the side-chain-protecting groups, except for cysteines with Acm. Then, the cleavage mixture was filtered and precipitated with 40 mL of cold ether. The crude peptide was then precipitated by centrifugation at 9000× *g* for 15 min and washed twice with approximately 40 mL of cold ether, air-dried and then dissolved in 5 mL 0.05% TFA, 50% acetonitrile containing and vacuum lyophilized. The crude peptides were solubilized with 50 mL of HPLC buffer B, diluted to 10-fold volume prior to purification by Semi-preparative RP-HPLC, using a preparative XBridge Peptide BEH C18 column eluted with a linear gradient ranging from 2 to 50% buffer B in 48 min at a flow rate 12 mL/min. The buffers were 0.05% (*v/v*) TFA in water (buffer A) and 0.05% TFA (*v/v*) in 60% aqueous acetonitrile (*v/v*) (buffer B). The eluent was monitored by measuring absorbance at 214 nm. The purity of the peptide was assessed by ACQUITY UPLC BEH C18 column RP-HPLC (buffer A: 0.05% (*v/v*) TFA in water; buffer B: 90% aqueous acetonitrile (*v/v*)) using the same gradient as described above with a flow rate 0.5 mL/min. The peptide solution with higher purity was collected, put into a −80 °C ultra-low temperature refrigerator overnight, and then frozen into powder by vacuum freeze-dryer. The linear peptides were then dissolved in 0.1 M Tris-HCl buffer containing 20 mM potassium ferricyanide to facilitate the formation of the first disulfide. The second disulfide bond was formed through iodine oxidation, as previously described [32]. The purity of RgIA and RgIA[11r] was confirmed using analytical RP-UPLC as described for the linear peptide after oxidative folding. Finally, electrospray mass spectrometry was utilized to confirm the molecular weight of the synthetic peptides. The purity for all fully folded peptides was ≥95% and was determined using analytical RP-UPLC.

### 4.3. cRNA Preparation and Injection into Xenopus laevis Oocytes

The plasmids containing the human, rat and mouse nAChR subunits were linearized using an appropriate restriction enzyme (TaKaRa, Kyoto, Japan). Capped RNA was synthesized in vitro for these subunits using the T7 mMessage mMachine Transcription Kit (Ambion, Austin, TX, USA) and purified using the MEGA clearTM Transcription clean-up Kit (Invitrogen; Thermo Fisher Scientific, Inc., Austin, TX, USA). The concentration of each cRNA was determined at 260 nm using a Smart SpecTM plus Spectrophotometer (Bio-Rad, Hercules, CA, USA). To produce 200–500 ng/μL of cRNA for each subunit, the various subunit cRNAs were mixed with ratio 1:1, and 50 nL aliquots were injected into the cytoplasm of the oocytes using a Drummond microdispenser (Drummond Scientific, Broomall, PA, USA). The oocytes were then incubated at 18 °C in ND-96 buffer (96 mM NaCl, 2 mM KCl, 1.8 mM CaCl_2_, 1 mM MgCl_2_ and 5 mM HEPES, pH 7.4) containing 10 mg/L penicillin, 10 mg/L streptomycin and 100 mg/L gentamicin.

### 4.4. Electrophysiological Recordings

As previously reported [33], *Xenopus* oocytes were analyzed using the two-electrode voltage-clamp method 3–5 days after injection. The oocytes were recorded using an Axoclamp 900A amplifier (Molecular Devices Corp., Sunnyvale, CA, USA) and the Clampfit 10.2 software (Molecular Devices Corp., Sunnyvale, CA, USA). The oocyte chamber was a cylindrical groove with a volume of approximately 50 mL, which was perfused with ND-96 buffer containing 0.1 mg/mL BSA at a flow rate of 2–4 mL/min under gravity. During the recording, the oocytes were held at a voltage of −70 mV and stimulated with a one-second pulse of 200 μM ACh every minute to stimulate α7 nAChRs. After establishing a stable baseline, the flow of buffer was stopped, and the oocytes were pre-incubated for 5 min with pure ND-96 or ND-96 containing different concentrations of conotoxins before the recovery of the ACh pulse.

### 4.5. Statistical Analysis

The percentage of response to conotoxin was obtained by dividing the toxin response by the pre-intoxication baseline value. Dose–response data were analyzed using GraphPad Prism 6.0 software (GraphPad Software, San Diego, CA, USA) and fitted with the formula: percentage response = 100/(1 − ([toxin]/IC_50_)^nH), where nH is the Hill coefficient. Each data point on the dose–response curve represents the mean ± S.E. of at least three oocytes. The IC_50_ values with 95% confidence interval were determined by nonlinear regression analysis with GraphPad Prism software.

### 4.6. Circular Dichroism (CD) Spectroscopy

CD spectra of RgIA and RgIA[11r] in aqueous solution were obtained using a Chirascan CD spectrometer (Applied Photophysics, Leatherhead, UK). The CD spectra were recorded at room temperature under a constant nitrogen flush. The peptides were dissolved in water at a concentration of 200 µM and measurements were performed in a 1 mm quartz cuvette. Peptide spectral data were recorded in the far UV range (190–260 nm) with a step size and bandwidth of 1 nm. The spectra were obtained from the average of five measurements after subtraction of the background signal, which was also averaged. The spectra were expressed in units of molar ellipticity (θ) using Applied Photophysics Chirascan and Photophysics Chirascan software (Applied Photophysics, Leatherhead, U.K.).

### 4.7. Serum Stability Assay

Serum stability assays were conducted employing human serum sourced from male AB plasma (Sigma-Aldrich, Darmstadt, Germany) following a modified protocol as previously described by Andrew et al. [34]. Human serum was subjected to an additional incubation period of 15 min at 37 °C prior to the commencement of the assay. Triplicate samples of peptides were dissolved in Milli-Q water at a concentration of 1 mg/mL and subsequently diluted with serum to a concentration of 0.1 mg/mL. The samples were then incubated at 37 °C, and 20 μL aliquots were collected at specific time intervals of 0, 1, 5, 10, 15, 30, 60, 120, 240 and 480 min. Each sample underwent denaturation by adding an equal volume of 6 M urea and incubating at 4 °C for 10 min. Following denaturation, precipitation was achieved by adding an equal volume of 20% trichloroacetic acid at 4 °C for 10 min. The samples were subsequently centrifuged at 14,000 rpm for 10 min, and 10 μL of the resulting supernatant was analyzed using RP-UPLC. Each sample was subjected to repeated analysis twice. The remaining percentage of the peptide was determined by comparing the areas under the curve to the samples measured at the initial time point (0 min).

### 4.8. Docking

The glide docking method was employed to construct the ligand–receptor complex. As a template, the cocrystal structure of RgIA and RgIA[11r] in complex with the α7 (PDB: 7EKT) was utilized. The three-dimensional structures of RgIA were retrieved from the RCSB database (PDB: 2JUT). Subsequently, the native RgIA was subjected to RgIA[11r] using Schrödinger software(2020, New York, USA). The dimensions of the simulation box were set as ligand of template. The docking procedure utilized the standard precision (SP) method for docking precision and employed a flexible ligand sampling approach. Specifically, 50 poses per ligand were generated. To assess the binding affinity of each ligand, the docking score calculation was employed to determine the potency of each pose [35,36].

## 5. Conclusions

In this study, the D-type amino acid RgIA[11r] mutant of RgIA lost its inhibitory activity at the α9α10 nAChRs, but gained the ability to selectively target and inhibit both human and rat α7 nAChRs, while not inhibiting other subtypes of nAChRs. The pharmacological profile of RgIA[11r] renders it a promising molecular probe for studying α7 nAChRs, and it also demonstrates the potential for therapeutic applications. In addition, the findings from this study suggest that D-type amino acid substitution can modify not only peptide stability, but also peptide activity. Therefore, it represents an effective strategy for peptide modification, and has the potential to lead to the discovery of novel peptide-based therapeutics with improved properties.

## Figures and Tables

**Table 1:** Inhibition of rat and human α7 and α9α10 nAChRs by RgIA and RgIA[11r].

Receptors	RgIA			RgIA[11r]			IC_50_ Ratio of RgIA/RgIA[11r]
	IC_50_ (95% CI *) (nM)	Hill Slope	*n*	IC_50_ (95% CI *) (nM)	Hill Slope	*n*	
rα7	5313 (3068–9202)	0.8 (0.4–1.1)	3	163 (143–187)	0.8 (0.7–0.9)	11	32.6
hα7	4608 (2516–8440)	0.7 (0.4–1.0)	4	463 (333–640)	0.6 (0.5–0.7)	7	9.96
rα9α10	2.6 (2.1–3.2)	0.8 (0.7–0.9)	11	15,820 (10,380–24,200)	0.8 (0.5–1.2)	6	0.00016
hα9α10	1398 (997–1961)	0.5 (0.4–0.6)	6	>10,000	-	3	-

* IC_50_ values with 95% confidence interval; Hill slope obtained from the concentration−response curves for RgIA and RgIA[11r] at α7 nAChRs. All data represent mean ± SEM of *n* = 3−11 experiments.

**Table 2:** Inhibition of α7 nAChRs by α- conotoxins.

Name	Sequences	α7 (IC_50_, nM)	Other nAChR Subtypes	Ref.
RgIA[11r]	GCCSDPRCRYrCR	rα7 (163) > hα7 (463)	>10,000	This work
RegIIA	GCCSHPACNVNNPHIC #	rα7 (41) > hα7 (210)	α3β2 > α3β4 > α7 α6/α3β4β3	[20,21]
[H5D]RegIIA	GCCSDPACNVNNPHIC #	rα7 (100) > hα7 (13,680)	N.D.	[20]
OmIA	GCCSHPACNVNNPHICG #	rα7 (59) > hα7 (290)	α3β2 > αα6/α3β2 > α7	[22]
PnIA	GCCSLPPCAANNPD(sTy)C #	hα7-5HT3 chimaera (510), rα7 (252)	α3β2 > α7	[23,24]
PnIA [A10L,sTy15Y]	GCCSLPPCALNNPDYC #	rα7 (12) > hα7 (59)	α3β2 ≈ α7	[20]
[Q1G,ΔR14]LvIB	GCCSNPPCAHEHC #	rα7 (97) > hα7 (1570)	α7 > α6/α3β2β3 > rα3β2 > rα6/α3β4	[19]
ImI	GCCSDPRCAWRC #	rα7 (69) > hα7 (440)	hα3β2 ≈ α7	[17,18]
ImII	ACCSDPRCAWRC #	rα7 (441) > hα7 (571)	α7 > α1β1δε	[17]
LsIA	SGCCSNPACRVNNPNIC #	hα7 (10.1)	α7 ≈ α3β2 > α3α5β2	[25]
ArIA	IRDECCSNPACRVNNPHVCRRR	rα7 (6.02)	α7 >α3β2	[24]
ArIB	DECCSNPACRVNNPHVCRRR	rα7 (1.81)	α7 > α6/α3β2β3 > α3β2	[24]
ArIB [V11L, V16A]	DECCSNPACRLNNPHACRRR	rα7 (0.356)	α7 > α6/α3β2β3 > α3β2	[24]
EpI	GCCSDPRCNMNNPD(sTy)C #	rα7 (30)	α7 > α3β4	[26,27]
GID	IRD(Gla)CCSNPACRVNNOHVC	hα7 (4.5) > rα7 (5.1)	α3β2 > α7 > α3β4	[28,29]

sTy refers to sulfotyrosine; O is hydroxyproline; Gla is γ-carboxyglutamic acid; # denotes C-terminal carboxamide.

## Data Availability

The data presented in this study are available in the article.
